# Contribution of the GABAergic System to Non-Motor Manifestations in Premotor and Early Stages of Parkinson’s Disease

**DOI:** 10.3389/fphar.2019.01294

**Published:** 2019-10-30

**Authors:** Ane Murueta-Goyena, Ane Andikoetxea, Juan Carlos Gómez-Esteban, Iñigo Gabilondo

**Affiliations:** ^1^Neurodegenerative Diseases Group, Biocruces Bizkaia Health Research Institute, Barakaldo, Spain; ^2^Department of Neuroscience, University of the Basque Country (UPV/EHU), Leioa, Spain; ^3^IKERBASQUE Basque Foundation for Science, Bilbao, Spain

**Keywords:** GABA, REM sleep behavior disorder, hyposmia, visual alterations, gastrointestinal symptoms, neurotransmitters

## Abstract

Non-motor symptoms are common in Parkinson’s disease (PD) and they represent a major source of disease burden. Several non-motor manifestations, such as rapid eye movement sleep behavior disorder, olfactory loss, gastrointestinal abnormalities, visual alterations, cognitive and mood disorders, are known to precede the onset of motor signs. Nonetheless, the mechanisms mediating these alterations are poorly understood and probably involve several neurotransmitter systems. The dysregulation of GABAergic system has received little attention in PD, although the spectrum of non-motor symptoms might be linked to this pathway. This Mini Review aims to provide up-to-date information about the involvement of the GABAergic system for explaining non-motor manifestations in early stages of PD. Therefore, special attention is paid to the clinical data derived from patients with isolated REM sleep behavior disorder or drug-naïve patients with PD, as they represent prodromal and early stages of the disease, respectively. This, in combination with animal studies, might help us to understand how the disturbance of the GABAergic system is related to non-motor manifestations of PD.

## Introduction

Parkinson’s disease (PD) is the second most common neurodegenerative disorder with a prevalence of between 1% and 4% in over-60-year-olds (Tysnes and Storstein, 2017). The diagnosis of PD currently depends on the identification of motor clinical features, including rest tremor, rigidity and bradykinesia. In addition, patients with PD develop a wide range of non-motor manifestations including cognitive impairment and dementia, mood and sleep disturbances, sensory abnormalities and autonomic nervous system dysfunction (Poewe, 2008). It has been estimated that the first motor signs appear when 50% to 80% of dopaminergic neurons in the substantia nigra pars compacta have been lost (Cheng et al., 2010). Thus, by the time of diagnosis, brain injury has been ongoing for years and any attempt at neuroprotection at this stage might be unsuccessful. Great efforts are being made to detect markers of neuronal dysfunction early in the course of the disease (Postuma and Berg, 2019). In relation to this, it is increasingly recognized that non-motor symptoms not only accompany but also precede motor signs in PD (Poewe, 2008). This is consistent with the Braak PD staging system, which suggests that α-synuclein deposition starts in areas involved in sleep regulation, olfaction or autonomic function before affecting the basal ganglia or cerebral cortex (Braak et al., 2003). The array of premotor symptoms might help to identify patients at high risk of developing α-synuclein-mediated neurodegenerative diseases, such as PD, dementia with Lewy bodies (DLB) or multiple system atrophy (MSA).

**Figure 1:**
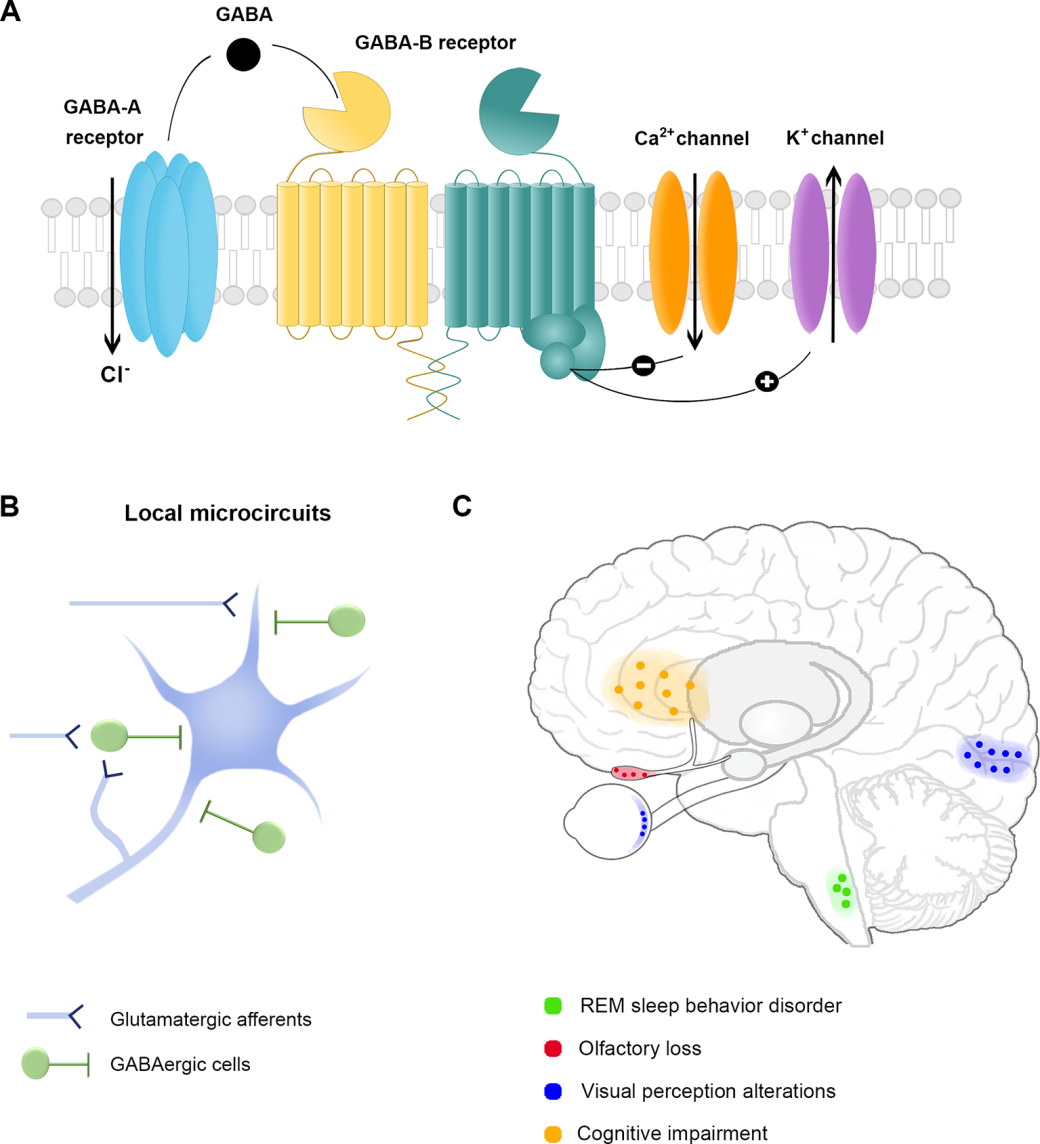
The GABAergic system and non-motor symptoms in Parkinson’s Disease. **(A)** GABA receptors. The inhibitory neurotransmitter GABA acts through ionotropic GABA_A_ receptor or metabotropic GABA_B_ receptor to reduce the membrane potential. The activation of GABA_A_ receptors allows chloride (Cl^-^) entry into the cytoplasm, while GABA_B_ receptor activation leads to a cellular cascade resulting in calcium (Ca^2+^) channel deactivation and potassium (K^+^) channel opening. **(B)** Schematic representation of cortical and subcortical local microcircuit organization of GABAergic cells. Inhibitory GABAergic cells are primarily local projecting neurons with a broad array of anatomical and physiological properties. The effect resulting from the inhibition exerted by GABAergic cells depends on their sensitivity to incoming stimuli, their firing properties and the subcellular domain of excitatory cells targeted by each interneuron. The diversity of GABAergic cells provides the brain with extensive computational power to regulate sensory and cognitive processes. **(C)** Brain areas associated with non-motor symptoms in Parkinson’s disease. Each color corresponds to a specific non-motor symptom and the associated area of the presumed GABAergic dysfunction.

While the mechanisms for motor impairment are fairly well established, the neuroanatomical and molecular substrates for non-motor manifestations are far from clear. Current evidence suggests that neurotransmitters, such as acetylcholine, serotonin, noradrenaline, glutamate and gamma-aminobutyric acid (GABA), play an important role in the pathophysiology of PD (Sanjari Moghaddam et al., 2017). GABA is the main inhibitory neurotransmitter in the central nervous system (CNS), acts through GABA_A_ and GABA_B_ receptors, and is primarily released by local interneurons to regulate cortical and subcortical microcircuits (**Figures 1A, B**). GABAergic signaling modulates a wide range of physiological functions, including sensory perception, information processing and cognition. In patients with PD, GABAergic dysregulation has been observed in the basal ganglia *postmortem* and *in vivo* with magnetic resonance spectroscopy (Kish et al., 1986; Emir et al., 2012; O’Gorman Tuura et al., 2018). Recently, it has been shown that striatal dopaminergic axons co-release GABA (Tritsch et al., 2012; Tritsch et al., 2014), which suggests that dopaminergic neurodegeneration could lead to GABA decline in basal ganglia circuits (O’Gorman Tuura et al., 2018). Considering that GABAergic networks regulate calcium-mediated mechanisms, like mitochondrial function and oxidative stress, loss of GABA inhibitory tone would facilitate accumulation of abnormal levels of intracellular calcium, triggering neurodegenerative processes. Consistent with this idea, it has been shown that GABA agonists, such as Baclofen or Bumetadine, relieve motor symptoms and protect dopaminergic cell bodies in mice models of PD (Hajj et al., 2015; Lozovaya et al., 2018). Nonetheless, GABAergic alterations might go beyond the basal ganglia. Unfortunately, few studies have investigated how GABAergic or other neurotransmitter systems may induce or modulate non-motor symptoms of PD. Identifying the separate role of each pathway may allow us to develop novel pharmacological compounds targeted to specific symptoms. Seeking to provide up-to-date information about the role of the GABAergic system, in this Mini Review, we focus on its ability to explain some of the non-motor manifestations that appear early in PD (**Table 1**).

**Table 1:** Evidence of the involvement of GABAergic neurotransmission in early non-motor symptoms of Parkinson’s disease.

Non-motor symptom	Evidence of GABAergic inhibitory deficit
REM sleep behavior disorder	GABAergic cells in the ventral medulla are dysfunctional ^†^ (Brooks and Peever, 2011)
Olfactory loss	Tonic inhibition exerted by interneurons regulates odor detection (Pirez and Wachowiak, 2008; Shao et al., 2009; Acebes et al., 2011)
Visual perception alterations	GABAergic depletion in the retina changes contrast sensitivity ^†^ (Hilgen et al., 2015)
	GABA antagonism in visual cortex decreases stimulus orientation and direction selectivity ^†^ (Katzner et al., 2011)
	GABA levels in visual cortex are predictive of visuospatial abilities * (Cook et al., 2016)
	Visual hallucinations are associated with decreased occipital GABA in PD (Firbank et al., 2018) and with the loss of postsynaptic GABA markers in DLB ** (Khundakar et al., 2016)
Cognitive impairment	GABA transcriptional changes in the frontal cortex ** (Santpere et al., 2018)
	Levels of PV and GAD67 mRNA expression are low in the frontal cortex ** (Lanoue et al., 2013; Lanoue et al., 2010)
Anxiety and depression	GABA_A_ receptor positive modulators are anxiolytic and antidepressant, while negative modulators produce anxiogenic and depressive-like effects ^†,*^ (Kalueff and Nutt, 2007; Mohler, 2012)
	GABA receptor dysfunction is linked to anxiety and depression-like behaviors ^†^ (Kalueff and Nutt, 2007)
Gastrointestinal symptoms	GABA regulates the mobility and inflammatory responses of the gastrointestinal tract ^#^ (Auteri et al., 2005a; Auteri et al 2005b; Jin et al., 2013)

^†^Studies conducted in animal models, ^#^physiological function, * in healthy controls, ** in patients with Parkinson’s disease (PD) or dementia with Lewy bodies (DLB).

## REM Sleep Behavior Disorder and Pontine GABAergic Cell Dysfunction in PD

Sleep disturbances are the most common non-motor manifestations of PD, with wide variability in the reported prevalence (66% to 98%) (Garcia-Borreguero et al., 2003). Sleep-related abnormalities in PD include insomnia, sleep fragmentation, restless legs syndrome, excessive daytime sleepiness and rapid eye movement (REM) sleep behavior disorder (RBD), among others. Some sleep abnormalities occur in early stages of the disease, even during the prodromal phase, including RBD (Iranzo et al., 2006), restless legs syndrome (Wong et al., 2014) and excessive daytime sleepiness (Abbott et al., 2005). Nonetheless, only idiopathic RBD (iRBD) has consistently shown to be an early predictor of the development of PD (Iranzo et al., 2017) (Postuma and Berg, 2019).

RBD is a parasomnia characterized by the loss of the normal muscle atonia of REM sleep. The diagnostic hallmark is excessive electromyographic activity during REM sleep as documented by polysomnography (Ferini-Strambi et al., 2016). Patients with RBD report the enactments of dreams, including kicking, punching or talking. In the absence of other neurological signs or CNS lesions, patients with iRBD are at high risk of developing α-synuclein-mediated neurodegenerative diseases in the years following the diagnosis (Hogl et al., 2018). It has also been suggested that the presence of RBD in PD patients is associated with an aggressive phenotype, these patients showing a higher density of α-synuclein aggregates (Knudsen et al., 2018).

Therefore, in recent years, special attention has been paid to PD-related non-motor manifestations and symptom progression in iRBD patients, seeking to find novel biomarkers of PD. Although the precise pathophysiological mechanisms for iRBD have not been fully determined, iRBD seems to be attributable to neurochemical imbalances in sleep regulatory systems (Boucetta et al., 2014). Previous studies have pointed toward a significant and specific neurodegeneration of GABA or glycine-containing neurons in the ventral medulla, such as in the nucleus raphe magnus and the ventral gigantocellular, alpha gigantocellular and lateral paragigantocellular reticular nuclei that directly project to spinal motor neurons to produce atonia during REM sleep (Iranzo, 2018) (**Figure 1C**). This hypothesis is supported by preclinical studies in transgenic mice that exhibit an RBD phenotype when glycine and GABA receptor function is impaired (Brooks and Peever, 2011). Moreover, allosteric agonists that bind at the α/γ subunit interface of GABA_A_ receptors, i.e., benzodiazepines, including clonazepam, triazolam or alprazolam, are the first-line therapy in iRBD (Anderson and Shneerson, 2009), and the effectiveness of this treatment could be explained by GABAergic neurotransmission disruption in prodromal stages of PD.

## Olfactory Loss and Its Relationship With GABAergic Neurotransmission in PD

Olfactory dysfunction is observed in more than 90% of PD patients (Doty, 2012), frequently precedes the onset of motor symptoms (Fantini et al., 2006; Ross et al., 2008; Postuma et al., 2009), and predicts the early conversion of iRBD to PD or DLB (Mahlknecht et al., 2015; Fereshtehnejad et al., 2017). The mechanisms responsible for olfactory dysfunction in PD are currently unknown. Magnetic resonance imaging studies have shown significantly smaller olfactory bulb volumes in patients with PD than controls (Brodoehl et al., 2012; Li et al., 2016; Tanik et al., 2016), although other authors failed to find such differences (Altinayar et al., 2014). Axonal and myelin damage of olfactory tracts has also been observed using diffusor-tensor imaging (Scherfler et al., 2006; Scherfler et al., 2013). These results have been confirmed by *postmortem* analysis of olfactory bulbs, in which global glomerular voxel volume was found to be smaller in five PD cases than six healthy controls (Zapiec et al., 2017). Moreover, hyposmia has been related to pathological changes in other areas of the olfactory system, such as the anterior olfactory nucleus or basolateral nucleus of the amygdala (Pearce et al., 1995; Harding et al., 2002). On the other hand, sensory perception disturbances might represent subtle alterations of normal functioning that precede neuronal degeneration. Changes in network connectivity of brain structures related to olfaction have already been described (Westermann et al., 2008; Bohnen et al., 2010; Wen et al., 2017), and these functional abnormalities may arise from iron and sodium deposition (Gardner et al., 2017).

The limited literature about the precise anatomy and physiology of the human olfactory bulb makes it difficult to assess the mechanisms related to olfactory dysfunction in humans. In this regard, animal studies provide a wealth of knowledge, as the olfactory bulb of rodents has been well characterized. It has been shown that interneurons—GABA-releasing cells—are essential for odor detection, and functionally distinct GABAergic circuits within the olfactory bulb of rodents play different roles in olfactory coding. The tonic inhibition exerted by these cells is thought to regulate the sensitivity of odor detection and odor perception in the mammalian brain (Pirez and Wachowiak, 2008; Shao et al., 2009; Acebes et al., 2011) (**Figure 1C**). Even though animal findings suggest that interneuron connectivity is the major determinant of odor perception, whether the loss of inhibitory synapses contributes to olfactory changes in PD in humans needs further research.

## Visual Disturbances

Among primary visual functions, low-contrast visual acuity, contrast sensitivity and color vision are typically affected in PD (Weil et al., 2016). Patients with drug-naïve PD or iRBD also show decreased contrast sensitivity (Righi et al., 2007; Marques et al., 2010), and abnormal color vision discrimination has been described in iRBD, these patients having a 3-fold higher risk of conversion (Postuma et al., 2015). Nevertheless, color discrimination is not consistently impaired in the early stages of PD, indicating that color vision abnormalities may represent a specific PD phenotype (Vesela et al., 2001). Indeed, Postuma and colleagues reported that abnormal color vision in iRBD was a stronger predictor of primary dementia than parkinsonism (Postuma et al., 2015), which is in line with findings in PD, RBD increasing the risk of cognitive decline (Pagano et al., 2018). Patients with iRBD or *de novo* PD also display visuoconstructional and visuoperceptual disturbances that may be related to non-dopaminergic impairment (Ferini-Strambi et al., 2004; Gagnon et al., 2009; Aarsland et al., 2009a; Marques et al., 2010; Fantini et al., 2011; Kim et al., 2011; Ota et al., 2016).

*In vivo* neuroimaging studies in newly diagnosed and drug-naïve PD patients have detected structural alterations in the visual pathway, ranging from thinning of inner retinal layers to increased optic radiation mean diffusivity and reduced visual cortical volumes (Arrigo et al., 2017; Ahn et al., 2018; Murueta-Goyena et al., 2019), which might explain some visual disturbances. There is, however, a growing body of evidence highlighting the role of GABA in perceptual aspects of vision.

Retinal amacrine cells co-release dopamine and GABA and the degeneration of these specialized cells has been suggested to cause primary visual dysfunction, although this hypothesis has not been confirmed (Nguyen-Legros, 1988). In line with this, pharmacological depletion of endogenous retinal GABA with allylglycine induces changes in contrast sensitivity (Hilgen et al., 2015). On the other hand, animal studies have shown that GABA_A_ receptor antagonist infusion in cat primary visual cortex decreases selectivity for stimulus orientation and direction, but not contrast sensitivity (Katzner et al., 2011). More recently, it has been observed that GABA levels measured by magnetic resonance spectroscopy are strong predictors of visuospatial abilities in healthy adults (Cook et al., 2016), and increasing GABA activity with systemic midazolam injections decreases visual sensitivity, preferentially affecting medium-to-high spatial frequencies and low temporal frequencies (Blin et al., 1993). Additionally, higher GABA concentrations in the visual cortex, as well as administration of the GABA agonist lorazepam, induce slower perceptual dynamics (van Loon et al., 2013).

These findings suggest that GABA signaling plays a central role in visual perception and a disturbance of this circuit at any level of the visual pathway could influence proper sensory processing. Consistent with this, recent studies show that PD patients with visual hallucinations have low occipital GABA concentrations (Firbank et al., 2018), and complex visual hallucinations in DLB are associated with altered GABAergic synaptic activity (Khundakar et al., 2016), which further supports the view that dysregulation of GABAergic system is involved in the visual pathway of PD (**Figure 1C**). Whether this system is affected in the retina and visual cortex of all PD patients, and from early stages, remains to be determined.

## Cognitive Dysfunction and GABAergic Signaling in Frontostriatal Circuits

Cognitive manifestations are frequently reported in PD, with a prevalence of 20–25% for mild cognitive impairment and 30% for dementia. It is estimated that PD patients have a 3- to 6-fold higher risk of developing dementia than age-matched controls (Svenningsson et al., 2012). Cognitive dysfunction is thought to be one of the key premotor manifestations of PD. At diagnosis, 15–20% of PD patients have mild cognitive impairment (Aarsland et al., 2009b) and several studies in patients with iRBD have identified cognitive disturbances, including delayed verbal memory, poorer decision-making, worse attention and slower processing speed, these domains being predictive of future risk of developing PD or DLB (Fantini et al., 2011; Terzaghi et al., 2013; Youn et al., 2016; Genier Marchand et al., 2017). Thus, early onset cognitive abnormalities are mainly dependent on the frontal lobe (Ferini-Strambi et al., 2004; Massicotte-Marquez et al., 2008; Sasai et al., 2012; Chahine et al., 2018).

Despite the evidence of neuropathological abnormalities in frontal brain areas in PD, their molecular and cellular alterations are poorly understood. Several studies have suggested that cognitive impairment in PD is attributable to neurotransmitter dysregulation rather than frank neurodegeneration (Kehagia et al., 2010; Ray and Strafella, 2012). Dopamine and acetylcholine deficiencies in frontostriatal pathways play a major role in cognitive impairment in PD, but the contribution of the other neurotransmitter systems remains less certain. Regarding the role of GABA, in frontal cortex global transcriptional changes of GABAergic neurotransmission have been observed in DLB patients (Santpere et al., 2018). It has also been shown that mRNA expression of the GABA-synthesizing enzyme glutamic acid decarboxylase-67 (GAD67) (Lanoue et al., 2010) and the calcium-binding protein parvalbumin (PV) (Lanoue et al., 2013)—two key markers of GABAergic cells—is low in the dorsolateral prefrontal cortex of PD patients without evidence of cell loss, further suggesting the downregulation of inhibitory neurotransmission in the frontal cortex (**Figure 1C**). In basal ganglia, *in vivo* GABA concentration changes have been detected in PD patients performing cognitive tasks (Buchanan et al., 2015). Interestingly, boosting GABAergic neurotransmission by zolpidem administration in early stage PD patients modulates aberrant beta-frequency oscillations (Hall et al., 2014), and the desynchronization of low-frequency activity seems to restore cognitive functions (Hall et al., 2010). Although these findings point towards decreased GABAergic activity in frontostriatal circuits in PD and DLB, whether GABAergic neurotransmission is also perturbed in premotor stages of PD has not been established, and its contribution to cognitive dysfunction needs to be elucidated in future studies.

## GABA in Anxiety and Depression

Anxiety and depression are common non-motor symptoms of PD, with reported prevalence rates of 20–40% (Chen and Marsh, 2014) and 50% (Reijnders et al., 2008), respectively, and may precede motor signs (Jacob et al., 2010). Notably, RBD patients score worse on anxiety and depression scales than controls and even PD patients (Barber et al., 2017). Although the exact neurobiological mechanisms that underlie anxiety and depression have not been fully elucidated, they seem to be intrinsically interrelated.

Pharmacological studies in both humans and animals have revealed that positive modulators of GABA_A_ receptors are anxiolytic and antidepressant, whereas negative modulators produce anxiogenic and depressive-like effects (Kalueff and Nutt, 2007; Mohler, 2012). Agents that enhance GABA_A_ receptor conductance (e.g., benzodiazepines) and GABA metabolism (e.g., valproate, vigabatrin, and tiagabine) exert anxiolytic effects, and it seems that partial agonists of α_2_/α_3_ GABA_A_ receptors, such as TPA-023, may also serve as antidepressants (Mohler, 2012). Furthermore, genetic studies implicate GABA-receptor dysfunction in the risk of developing anxiety and depression (Kalueff and Nutt, 2007). Recent evidence suggest that somatostatin contributes to the pathology of anxiety and depression (Fuchs et al., 2016; Fee et al., 2017), levels of this GABAergic marker being low in cerebrospinal fluid and induced pluripotent cells of PD patients (Dupont et al., 1982; Iwasawa et al., 2019). Nonetheless, there is still a lack of studies exploring the role of somatostatin in anxiety and depressive disorders in PD.

## Gastrointestinal Symptoms and GABA Signaling in the Enteric Nervous System

Gastrointestinal disturbances fall within the spectrum of autonomic manifestations of PD patients. Hypersalivation, dysphagia, nausea, gastroparesis, small intestinal dysfunction, slow transit constipation and defecatory dysfunction have been attributed to α-synuclein-mediated small fiber neuropathy of the enteric nervous system (ENS) and to the neurodegeneration of the enteric branches of the vagus nerve in the brainstem (Pfeiffer, 2018). Among the gastrointestinal symptoms, constipation is the most frequent manifestation in PD and recent evidence suggests that it might also be one of the most common disturbances in prodromal PD (Stirpe et al., 2016). A multicenter study of 318 patients with polysomnography-confirmed iRBD concluded that they had substantially more autonomic symptoms than controls (SCOPA-AUT questionnaire), gastrointestinal symptoms being the most prominent domain (Ferini-Strambi et al., 2014). Nonetheless, gastric emptying measured with the 13C‐octanoate breath test showed that only drug‐naïve and early‐stage Parkinson’s disease patients had delayed gastric emptying, authors suggesting that changes in structures modulating gastric motility might not be sufficiently severe in iRBD (Unger et al., 2011).

The last three decades have seen an expansion in the literature on the role of GABA in the control of gastrointestinal function, including mobility and inflammatory responses (Auteri et al., 2015a; Auteri et al., 2015b). GABA has been identified as an important modulator of gastrointestinal tract function. This neurotransmitter can stimulate or inhibit the enteric neurons acting though GABA_A_ or GABA_B_ receptors (Auteri et al., 2015a). Its role is particularly important in the colon, where it modulates the peristatic reflex. On the other hand, enteric inflammation occurs in PD and has been related to the initiation and progression of the disease (Houser and Tansey, 2017). Nonetheless, it has yet to be determined why the production of pro-inflammatory cytokines takes place in the enteric tract. The purinergic system controls enteric inflammation, but GABA also has a major role in immune cell activity and inflammatory events in the gastrointestinal tract (Jin et al., 2013). Topiramate—an anti-epileptic drug that acts as a GABA_A_ agonist—reduces gastrointestinal inflammation in rats (Dudley et al., 2011), identifying GABA as a putative neuroimmune modulator. A better understanding of the relationship of GABA signaling with intestinal motility and inflammation is necessary, however, to reveal a possible functional link between this neurotransmitter, the ENS, and gastrointestinal symptoms of PD.

## Final Remarks and Conclusions

Current evidence supports the view that PD is a degenerative disorder that affects multiple systems and presents with several non-motor symptoms. Over recent years, the importance of early, non-motor manifestations of PD has been increasingly recognized, as they may help to identify patients at high risk of developing α-synucleinopathies. Even though the neuronal circuits for motor symptoms are fairly well understood, the pathophysiological mechanisms for perceptual, cognitive, mood and autonomic disturbances of PD remain unclear.

Here, we report evidence consistent with the view that the GABAergic system is altered in PD and may contribute to non-motor symptoms that appear early in disease progression. Nonetheless, the literature in this field is dominated by non-placebo controlled and *postmortem* studies, generally based on small series and providing low-level evidence. To summarize, based on current findings, PD patients in premotor stages have anxiety and depression and alterations in the olfactory system, visual perception and visuospatial abilities, frontostriatal-related cognition, and gastrointestinal function. The neurobiological correlates of these deficits are unclear, in part because of the complex dynamic interactions between several neurotransmitter systems. Still, the dysfunction of GABAergic neurons in ventral medullary reticular formation seems to be linked to RBD. Moreover, preclinical studies show the relevance of interneurons in odor detection and the causal role of GABA in anxiety and depressive disorders, but we are far from establishing whether this also occurs in PD. On the other hand, disturbance of GABA signaling by pharmacological compounds affects visual processing and cognition, and GABA levels in the visual cortex are low in PD patients with visual hallucinations. It has been also shown that GABA controls gastrointestinal function, although it is not known whether this is associated with the gastrointestinal symptoms reported by PD patients. All these findings suggest that intervening in GABAergic signaling might modulate non-motor manifestations of PD and provide a novel avenue for non-dopaminergic therapy.

Nevertheless, there is a paucity of replication and large case-control studies. Future research should include *in vivo* longitudinal studies that examine the link between alterations in the GABAergic system and early non-motor symptoms by exploiting advances in PET ligands, magnetic resonance spectroscopy and CSF biomarkers. Preclinical studies might help to investigate the effects of GABA in the pathogenesis of non-motor symptoms, but we suggest that identifying the neurotransmitter deficits that correlate with clinical severity should be the mainstay for guiding future treatment studies.

## Author Contributions

AM-G conceptualized and wrote the manuscript. AA organized and prepared the manuscript. JG-E and IG contributed to writing and reviewing the manuscript.

## Funding

This study was partially funded by the Michael J. Fox Foundation (2014 Rapid Response Innovation Awards; Grant 10189), the Carlos III Health Institute through Projects PI14/00679 and PI16/00005, and a Juan Rodes Grant (JR15/00008) (I.G.) (cofunded by the “Investing in Your Future” European Regional Development Fund/European Social Fund programme), the Department of Health of the Basque Government through Project 2016111009, and EITB/BIOEF telemarathon for Neurodegenerative Diseases (BIO17/ND/010/BC).

## Conflict of Interest

The authors declare that the research was conducted in the absence of any commercial or financial relationships that could be construed as a potential conflict of interest.
